# Cold-induced meiotic nondisjunction in *D. melanogaster*

**DOI:** 10.17912/micropub.biology.001439

**Published:** 2025-02-03

**Authors:** Lewis I. Held, Jr., Surya J. Banerjee, Souvik Roy, Hariz A. Nawaz, Jameson R. Hanes, Gal Ritte, Peyton N. Ansley, Lindsey K. McCurry, Karima H. Assal, Aubriana M. Benson, Ria Umbrani, Owen F. Vajnar, Kambre A. Huddleston, Lily M. Russell, Katharine A. Green, Natasha N. Guhl, Isaac B. Ireland, Estefania Ireland, Luis A. Rocha, Jamie F. Meuth, Sam P. Davis, Cassandra G. O'pry, Jason J. Shin

**Affiliations:** 1 Dept. of Biological Sciences, Texas Tech University, Lubbock, Texas, United States

## Abstract

Virgin *D. melanogaster* females were kept at near-freezing temperatures for up to one week, whereupon they were mated. Daily collections of offspring showed evidence of spindle malfunction in meiosis, but not in earlier mitoses, despite our usage of a colder temperature (5.5˚C) than previous studies (10˚C). The greater sensitivity of meiosis than mitosis is baffling, considering that the opposite is true for males, where primary spermatocytes are most vulnerable.

**Figure 1. Dominant mutations that were used to mark 3 rd chromosome homologs in this study:**
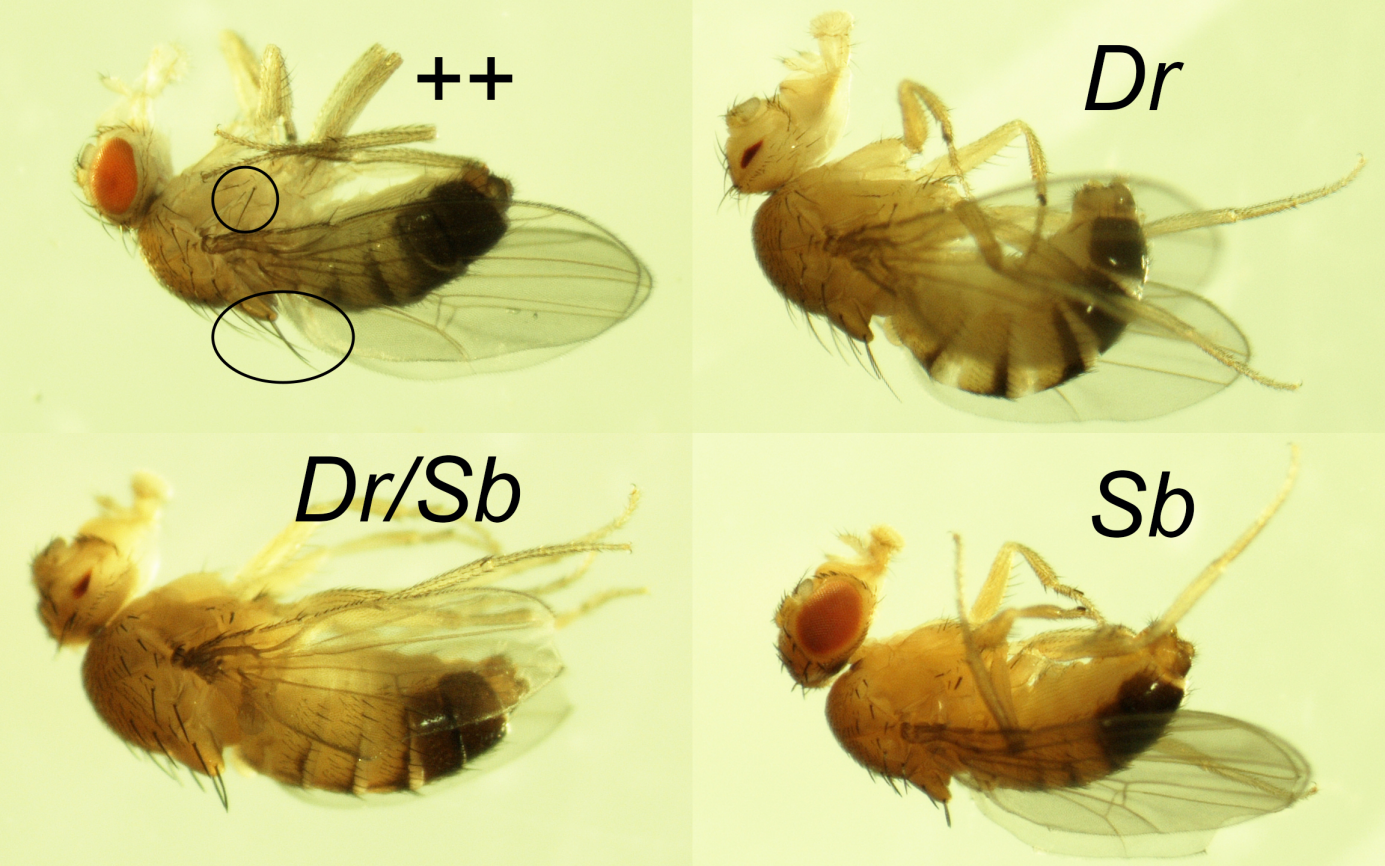
*Clockwise from upper left:* wild-type (++) Oregon R male (long, thin bristles on flank and back are circled), *Dr* male (slit-shaped eye), *Sb* male (truncated bristles), and *Dr/Sb* male-like intersex. The 3n intersex (lower left) was one of the 8 *Dr/Sb* F _1_ offspring from cold-treated *Dr/Sb* mothers in vial 7D (see Table 2). It has a larger thorax, and its *Sb* bristles are longer than those of the 2n *Sb* male (lower right), due to dose-dependence of the *Sb* phenotype (Lindsley and Zimm, 1992). All flies were photographed at the same magnification.

## Description

Temperatures near 0˚C inhibit polymerization of tubulin into microtubules (Inoué et al., 1974; Li and Moore, 2020), causing spindle malfunction (Steven et al., 2016) and chromosome nondisjunction (Woodruff and Thompson, 2002) . In 1970, Tokunaga showed that when virgin *D. melanogaster* females are kept at 10˚C for a week and then mated, ~5% of eggs laid on the first day become triploid (3n), implying that the oocytes had doubled in ploidy (1n to 2n) before fertilization (Tokunaga, 1970b) . However, 3n flies were only rarely recovered in later broods. Why? We sought to investigate this (and other) riddles by repeating and modifying her protocol.

**Riddle #1: Why are triploid offspring found only in the first brood?** Each of the two ovaries of a *D. melanogaster* female contains 15-20 tubular “ovarioles” (Hinnant et al., 2020) that function as “assembly lines” for egg production. At the apex of each such tube are 2-3 germline stem cells (GSCs) that divide asymmetrically. Their mitoses yield one daughter that remains a GSC and another that undergoes 4 more mitoses to form 16 cystocytes, one of which becomes the egg (Kirilly and Xie, 2007) . The cysts of cystocytes ratchet down the ovariole as they mature until they reach the oviduct, where the egg is later released (Bloch Qazi et al., 2003) . GSCs divide every 12-14 hours at 25˚C (Hinnant et al., 2020) . Because virgins arrest their eggs at metaphase of Meiosis I (Costa and Ohkura, 2019) , the fact that ploidy only increases in post-mating Brood #1 argues that *meiotic* spindles are vulnerable to cold, but *mitotic* spindles (of preceding cystocytes) are not. Indeed, Tokunaga (1970b) found that 10˚C only caused nondisjunction at Meiosis I, not at Meiosis II. *A priori* , one might have assumed that all spindles behave alike, but in *D. melanogaster* , meiotic spindles differ from mitotic spindles in architecture (Riparbelli and Callani, 2005) , polarity (Liang et al., 2009) , and other key features (Ohkura, 2015) , and mitotic spindles differ *inter se* in the construction devices they employ (Duncan and Wakefield, 2011) .

**Riddle #2: Are germline stem cells as insensitive to cold as cystocytes?** It takes 9-11 days from the GSC stage to a mature egg (Wieschaus and Szabad, 1979) , but Tokunaga did not examine broods beyond 11 days post-mating. Therefore, her data are moot as to whether GSCs are sensitive to cold, especially since cold shock might delay mitoses. If we could double the ploidy of GSCs (2n to 4n), then each such 4n GSC would yield a steady stream of 4n cystocytes that become 3n offspring in brood after brood, *ad infinitum* .

**Riddle #3: Is a full week required for cold treatment to affect spindles?** GSCs divide every 12-14 hours at 25˚C (Hinnant et al., 2020) , so why should a full week of cold exposure be needed to affect their spindles, assuming they are sensitive at all? Likewise, there is no obvious reason why a shorter treatment would not affect meiosis. Indeed, Tokunaga (1970a) showed that 3 days at 10˚C causes 0.59% meiotic nondisjunction (vs. 5.14% for 7 days of exposure and 0.03% for controls). Hence, we decided to try shorter durations as well.

It seemed possible that Tokunaga's failure to affect the ploidy of cystocytes was due to her usage of 10˚C instead of a lower (more effective?) temperature. Flies can withstand 4-5˚C for at least 4 days (Woodruff and Thompson, 2002) , so we decided to try 5.5˚C instead of 10˚C. Moreover, we examined F _1_ broods for 21 days (10 days longer than Tokunaga's span), which let us assess whether this stronger cold treatment can affect GSCs.

To detect ploidy-doubling events, we relied on an approach that one of us used long ago to look for 3n F _1_ from colcemid-treated fruit flies (Held, 1982) . In that study, 2 ^nd^ chromosomes were marked with dominant mutations ( *vg ^U^* and *Gla* ), one of which ( *Gla* ) was embedded in an inversion to prevent recovery of cross-overs. Here, the dominants are on 3 ^rd^ chromosomes instead: *Drop* ( *Dr* ) on one, and *Stubble* ( *Sb* ) on the other ( *TM3* balancer) homolog (Lindsley and Zimm, 1992) . *Dr* shrinks eyes, while *Sb* shortens bristles ( Fig. 1 ). Both are 100% penetrant and 100% expressed, thus affording 100% certainty in detecting 3n offspring that arise from retention of both markers in a single egg. Any *Dr/Sb* offspring must be triploids because aneuploidy for any long chromosome (2 or 3) by itself (isolated trisomy) is lethal (Ashburner, 1989) .

We exposed 4 cohorts of virgin *Dr* / *Sb* females (5 vials/cohort; 50 flies/vial) to 5.5˚C for 1, 2, 4, or 7 days, while controls were kept at 25˚C. After exposure, the *Dr/Sb* virgins were crossed with wild-type Oregon R males (25/vial) and transferred to fresh vials at 24 h intervals. All egg batches were allowed to develop fully, whereupon the F _1_ adults were preserved in 70% ethanol and screened for expression of both *Dr* and *Sb* . As explained above, *Dr/Sb* offspring must be 3n, and their frequency should reflect the effectiveness of 5.5˚C at blocking spindles. Table 1 tallies the offspring in Broods #1 and #2 for all 5 cohorts.

Table 1. Initial F _1_ broods after 0, 1, 2, 4, or 7 days of maternal 5.5˚C exposure.

**Table:** 

Cold duration	Brood #	Total F _1_	*Dr* only	*Sb* only	*Dr/Sb* *	% *Dr/Sb*
0 days (control)	1	3,522	1,872	1,650	0	0
"	2	3,615	1,736	1,879	0	0
1 day	1	3,839	2,087	1,752	3	0.1%
"	2	3,511	2,054	1,457	0	0
2 days**	1	369	219	150	2	0.5%
"	2	719	377	342	0	0
4 days	1	2,140	1,223	917	23	1.1%
"	2	3,031	1,741	1,290	0	0
7 days***	1	595	328	267	16	2.7%
"	2	3,310	1,887	1,423	0	0

* *Dr/Sb* F _1_ included 24 females (3X/3A?) and 20 male-like intersexes (2X1Y/3A?), where "3A" = 3 sets of autosomes. *Dr/Sb* F _1_ (3n) females are fertile.

**This cohort spent the first of the 2 days at 1˚C due to an incubator malfunction.

Hence, 56% (140/250) of the virgins died, drastically reducing the brood sizes.

***The reduced brood sizes for this cohort were not due to maternal mortality.

In agreement with Tokunaga (1970b), only Brood #1 yielded 3n F _1_ , with Brood #2 dropping to zero in every cohort. The frequency of 3n F _1_ increased with duration of exposure, reaching its peak in the 7-day cohort. While that rate (2.7%) was lower than Tokunaga's 5%, the 5 vials varied from 0.8% (1/123) for vial 7B to 5.8% (8/139) for vial 7D. In order to maximize our chance of finding 3n F _1_ from GSCs, we inspected 3 weeks of broods from vial 7D (Table 2).

Table 2. Daily batches of F _1_ offspring from vial 7D (exposed to 5.5˚C for 7 days).

**Table:** 

Stage affected	Brood #	Total F _1_	*Dr* only	*Sb* only	*Dr/Sb*	% *Dr/Sb*
Meiosis	1	139	72	67	8*	5.8%
Cystocytes	2	536	279	257	0	0
"	3	806	445	361	0	0
"	4	666	337	329	0	0
"	5	541	239	302	0	0
"	6	609	341	268	0	0
"	7	654	316	338	0	0
"	8	673	350	323	0	0
" or GSCs?	9	697	320	377	0	0
" or GSCs?	10	572	289	283	0	0
" or GSCs?	11	616	326	290	0	0
GSCs	12	545	280	265	0	0
"	13	438	256	182	0	0
"	14	579	276	303	0	0
"	15	307	114	193	0	0
"	16	408	198	210	0	0
"	17	446	276	170	0	0
"	18	317	171	146	0	0
"	19	346	189	157	0	0
"	20	312	144	168	0	0
"	21	336	168	168	0	0

* *Dr/Sb* F _1_ included 3 females (3X/3A?) and 5 male-like intersexes (2X1Y/3A?), where "3A" = 3 sets of autosomes.

We had thought we might impact the mitotic spindles of cystocysts and/or GSCs by using a lower temperature than Tokunaga (5.5˚C vs. 10˚C), but the absence of 3n F _1_ in Broods #2-21 indicates that this did not happen. Thus, the answer to Riddle #2 is yes, and to Riddle #3 is no, while Riddle #1 remains unsolved.

Why should mitotic spindles be impervious to cold? Microtubules can be either cold-labile or cold-stable, even in different tissues of the same animal (Ochoa et al., 2011) , and key proteins can protect cold-stable microtubules down to 4˚C (Delphin et al., 2012) . Moreover, *D. melanogaster* expresses 4 *alpha* -tubulin isoforms and 4 *beta* ones (Fyrberg and Goldstein, 1990) , and they may react differently to cold. Indeed, one *alpha* (67C) is found mainly in the cystocyte lineage, so the cold immunity of cystocytes and GSCs might lie in different tolerances of their tubulin monomers (Myachina et al., 2017) . In males, one of the *betas* is used uniquely in testes, which may explain why chilling shows nondisjunction in later broods for males than females—namely in Broods #7-8, which correspond to the primary spermatocyte stage (Tokunaga, 1971) .

The lingering mystery from our study is an evolutionary one. It makes sense that ectotherms like flies evolved protective devices for microtubules that enhance their survival in winter, but why should certain spindles, like the one for Meiosis I, have been left “out in the cold,” so to speak, to fend for themselves, given that errors can lead to maladapted aneuploids? The uniqueness of female meiosis may conceivably be related to the ability of its products (egg nucleus + polar bodies) to fuse during parthenogenesis to form 2n, 3n, or even 4n nuclei (Sperling et al., 2023) .

## Methods

Our protocol was as follows: (1) raise virgin *Dr/Sb* flies at 25˚C, (2) expose 250 virgin females (50/vial) to 5.5˚C for varying lengths of time, (3) return them to 25˚C, (4) mate them with wild-type Oregon R males (25/vial), (5) transfer the parents to fresh vials daily, and (6) screen each brood for the presence of F _1_ expressing both *Dr* and *Sb* . The frequency of *Dr/Sb* (3n) offspring/brood gives the frequency of ploidy doubling. Control females (250 *Dr/Sb* virgins @ 50/vial) were kept at 25˚C, mated with Oregon R males, and transferred to fresh vials daily. To ensure virginity of *Dr/Sb* mothers we sexed individuals from our *Dr/Sb* stock at the pharate adult stage and placed female pupae in food vials, where they later eclosed. We kept all cohorts at 25˚C for 3 days to “fatten up” before transferring them to our cold incubator (experimental series) or mating with wild-type males (controls). Food vials contained blue *Drosophila* Instant Medium, Plain Formula 4-24 (Carolina Biological Supply), plus a garnish of Fleischmann's live dry yeast that was activated by wetting. Several drops of water had to be added to the instant food daily to prevent parents from dying of thirst.

## Reagents

Our *Dr/Sb* stock was constructed by crossing virgin females from a multiply-balanced starter stock ( *y ^1^ w ^1^ ; wg ^Sp-1^ /CyO; Dr ^1^ /TM3, Sb ^1^* ) that was obtained from the Bloomington Stock Center (#59967) with wild-type (Oregon R) males. We then purged the subsequent F _1_ -F _3_ generations of any unneeded mutations (i.e., *y ^1^ , w ^1^ ; wg ^Sp-1^ ,* and *CyO* ), in order to maximize fecundity, since the starter stock was poorly fertile; the final stock was highly fertile. To ensure temperature constancy, we used Model 307 Fisher incubators, with pre-calibrated glass thermometers whose sensor bulbs were placed in the vial racks themselves, so as to provide accurate temperature readings from the most proximal possible locations.
