# Corrigendum: Cold-induced meiotic nondisjunction in *D. melanogaster*

**DOI:** 10.17912/micropub.biology.001575

**Published:** 2025-03-11

## Description

The ORCiD for Souvik Roy has been corrected to 0000-0002-8896-9902.

